# Supplementary material for: Modeling glioblastoma heterogeneity as a dynamic network of cell states
Source: Mol Syst Biol. 2021 Sep 16;17(9):e10105. doi: 10.15252/msb.202010105 (PMC8444284; doi:10.15252/msb.202010105)
Supplement: Supplementary file 5 — Source Data for Figure 3 [file MSB-17-e10105-s001.zip › Figure3A_sourcedata/GSEA_3065/nosigdb_state3.GseaPreranked.1622718426028/RICHARDS_NOWAKOWSKI_SCIENCE_2017_MGE_RG2_UPREG.html]

Details for gene set RICHARDS\_NOWAKOWSKI\_SCIENCE\_2017\_MGE\_RG2\_UPREG[GSEA]

|  || Dataset | state3 |
| Phenotype | NoPhenotypeAvailable |
| Upregulated in class | na\_neg |
| GeneSet | RICHARDS\_NOWAKOWSKI\_SCIENCE\_2017\_MGE\_RG2\_UPREG |
| Enrichment Score (ES) | -0.42417893 |
| Normalized Enrichment Score (NES) | -1.7761519 |
| Nominal p-value | 0.0 |
| FDR q-value | 0.006463598 |
| FWER p-Value | 0.087 |
Table: GSEA Results Summary

  

Fig 1: Enrichment plot: RICHARDS\_NOWAKOWSKI\_SCIENCE\_2017\_MGE\_RG2\_UPREG      
 Profile of the Running ES Score & Positions of GeneSet Members on the Rank Ordered List

  

| PROBE | GENE SYMBOL | GENE\_TITLE | RANK IN GENE LIST | RANK METRIC SCORE | RUNNING ES | CORE ENRICHMENT || 1 | BCAN |  |  | 0 | 0.883 | 0.0115 | No |
| 2 | CENPF |  |  | 4 | 0.619 | 0.0192 | No |
| 3 | PRKDC |  |  | 6 | 0.588 | 0.0268 | No |
| 4 | HSP90B1 |  |  | 7 | 0.563 | 0.0341 | No |
| 5 | PDIA6 |  |  | 8 | 0.549 | 0.0413 | No |
| 6 | CD63 |  |  | 26 | 0.480 | 0.0455 | No |
| 7 | PRDX4 |  |  | 41 | 0.455 | 0.0498 | No |
| 8 | HSPA5 |  |  | 43 | 0.447 | 0.0555 | No |
| 9 | PDIA3 |  |  | 55 | 0.419 | 0.0596 | No |
| 10 | MKI67 |  |  | 59 | 0.407 | 0.0646 | No |
| 11 | PON2 |  |  | 63 | 0.389 | 0.0693 | No |
| 12 | NASP |  |  | 85 | 0.360 | 0.0715 | No |
| 13 | FAT1 |  |  | 95 | 0.350 | 0.0750 | No |
| 14 | BSG |  |  | 98 | 0.347 | 0.0792 | No |
| 15 | SCD |  |  | 108 | 0.340 | 0.0826 | No |
| 16 | NES |  |  | 113 | 0.332 | 0.0865 | No |
| 17 | ATP1B3 |  |  | 114 | 0.330 | 0.0908 | No |
| 18 | SEMA5A |  |  | 121 | 0.324 | 0.0943 | No |
| 19 | SMC2 |  |  | 125 | 0.322 | 0.0981 | No |
| 20 | EMP2 |  |  | 135 | 0.315 | 0.1011 | No |
| 21 | SLC16A1 |  |  | 138 | 0.314 | 0.1050 | No |
| 22 | DNMT1 |  |  | 149 | 0.310 | 0.1078 | No |
| 23 | ILDR2 |  |  | 171 | 0.299 | 0.1092 | No |
| 24 | CHD7 |  |  | 172 | 0.298 | 0.1131 | No |
| 25 | SRRM2 |  |  | 175 | 0.297 | 0.1167 | No |
| 26 | JAM2 |  |  | 217 | 0.275 | 0.1154 | No |
| 27 | HELLS |  |  | 218 | 0.275 | 0.1190 | No |
| 28 | SMC1A |  |  | 231 | 0.271 | 0.1211 | No |
| 29 | RCN1 |  |  | 233 | 0.270 | 0.1245 | No |
| 30 | TNC |  |  | 249 | 0.264 | 0.1261 | No |
| 31 | PNN |  |  | 256 | 0.260 | 0.1288 | No |
| 32 | SMC4 |  |  | 265 | 0.255 | 0.1312 | No |
| 33 | NOTCH1 |  |  | 272 | 0.253 | 0.1337 | No |
| 34 | ATAD2 |  |  | 282 | 0.249 | 0.1359 | No |
| 35 | STAG2 |  |  | 304 | 0.242 | 0.1365 | No |
| 36 | SALL3 |  |  | 312 | 0.240 | 0.1388 | No |
| 37 | NCL |  |  | 333 | 0.234 | 0.1395 | No |
| 38 | CCDC14 |  |  | 334 | 0.234 | 0.1425 | No |
| 39 | BRCA1 |  |  | 339 | 0.232 | 0.1451 | No |
| 40 | CTNNB1 |  |  | 373 | 0.224 | 0.1440 | No |
| 41 | SFRP2 |  |  | 418 | 0.215 | 0.1415 | No |
| 42 | TMPO |  |  | 420 | 0.214 | 0.1442 | No |
| 43 | KIF15 |  |  | 428 | 0.213 | 0.1462 | No |
| 44 | MIS18BP1 |  |  | 448 | 0.210 | 0.1466 | No |
| 45 | ALG8 |  |  | 457 | 0.207 | 0.1483 | No |
| 46 | FKBP10 |  |  | 481 | 0.201 | 0.1482 | No |
| 47 | MCM4 |  |  | 482 | 0.201 | 0.1508 | No |
| 48 | CREB5 |  |  | 497 | 0.199 | 0.1517 | No |
| 49 | LDLR |  |  | 498 | 0.199 | 0.1543 | No |
| 50 | FDPS |  |  | 518 | 0.196 | 0.1546 | No |
| 51 | RRM1 |  |  | 537 | 0.194 | 0.1550 | No |
| 52 | NCAPD3 |  |  | 561 | 0.191 | 0.1547 | No |
| 53 | MMS22L |  |  | 645 | 0.179 | 0.1471 | No |
| 54 | MSH6 |  |  | 682 | 0.174 | 0.1450 | No |
| 55 | LIG1 |  |  | 691 | 0.171 | 0.1463 | No |
| 56 | AASS |  |  | 696 | 0.170 | 0.1480 | No |
| 57 | CDC5L |  |  | 697 | 0.170 | 0.1502 | No |
| 58 | AHI1 |  |  | 707 | 0.169 | 0.1514 | No |
| 59 | SLC35F1 |  |  | 711 | 0.168 | 0.1532 | No |
| 60 | FAM111A |  |  | 712 | 0.168 | 0.1554 | No |
| 61 | TMEM97 |  |  | 740 | 0.165 | 0.1543 | No |
| 62 | CCT5 |  |  | 746 | 0.165 | 0.1559 | No |
| 63 | RDX |  |  | 797 | 0.158 | 0.1519 | No |
| 64 | BLM |  |  | 811 | 0.156 | 0.1524 | No |
| 65 | FANCD2 |  |  | 813 | 0.156 | 0.1543 | No |
| 66 | PHGDH |  |  | 828 | 0.155 | 0.1546 | No |
| 67 | NOP56 |  |  | 829 | 0.155 | 0.1567 | No |
| 68 | RFC4 |  |  | 835 | 0.154 | 0.1581 | No |
| 69 | RBL1 |  |  | 844 | 0.153 | 0.1591 | No |
| 70 | MAD2L1 |  |  | 846 | 0.153 | 0.1610 | No |
| 71 | HNRNPA2B1 |  |  | 863 | 0.151 | 0.1610 | No |
| 72 | CYR61 |  |  | 873 | 0.150 | 0.1619 | No |
| 73 | RAD18 |  |  | 874 | 0.150 | 0.1639 | No |
| 74 | CDK1 |  |  | 915 | 0.147 | 0.1610 | No |
| 75 | CLSPN |  |  | 922 | 0.146 | 0.1622 | No |
| 76 | KNTC1 |  |  | 935 | 0.144 | 0.1626 | No |
| 77 | IQGAP2 |  |  | 943 | 0.143 | 0.1637 | No |
| 78 | MCM7 |  |  | 949 | 0.143 | 0.1649 | No |
| 79 | TROVE2 |  |  | 960 | 0.141 | 0.1656 | No |
| 80 | CENPC |  |  | 992 | 0.139 | 0.1636 | No |
| 81 | ORC6 |  |  | 1000 | 0.138 | 0.1646 | No |
| 82 | UTP20 |  |  | 1002 | 0.138 | 0.1663 | No |
| 83 | DYNC2H1 |  |  | 1014 | 0.137 | 0.1668 | No |
| 84 | TMEM123 |  |  | 1047 | 0.134 | 0.1647 | No |
| 85 | SPECC1 |  |  | 1048 | 0.134 | 0.1664 | No |
| 86 | TMX1 |  |  | 1053 | 0.134 | 0.1677 | No |
| 87 | PCDH17 |  |  | 1059 | 0.134 | 0.1688 | No |
| 88 | FANCI |  |  | 1092 | 0.131 | 0.1667 | No |
| 89 | CHAF1A |  |  | 1106 | 0.129 | 0.1668 | No |
| 90 | BRCA2 |  |  | 1122 | 0.128 | 0.1667 | No |
| 91 | PTPN13 |  |  | 1167 | 0.124 | 0.1630 | No |
| 92 | DGKH |  |  | 1182 | 0.123 | 0.1629 | No |
| 93 | RAD51AP1 |  |  | 1196 | 0.122 | 0.1629 | No |
| 94 | MCM10 |  |  | 1197 | 0.122 | 0.1645 | No |
| 95 | CCT3 |  |  | 1198 | 0.122 | 0.1661 | No |
| 96 | PABPN1 |  |  | 1273 | 0.117 | 0.1587 | No |
| 97 | CCND2 |  |  | 1286 | 0.116 | 0.1588 | No |
| 98 | SFRP1 |  |  | 1291 | 0.116 | 0.1598 | No |
| 99 | SRSF7 |  |  | 1328 | 0.113 | 0.1570 | No |
| 100 | NUSAP1 |  |  | 1338 | 0.113 | 0.1574 | No |
| 101 | SRSF10 |  |  | 1345 | 0.112 | 0.1581 | No |
| 102 | ESCO2 |  |  | 1373 | 0.110 | 0.1563 | No |
| 103 | LIMD1 |  |  | 1375 | 0.110 | 0.1576 | No |
| 104 | CNTLN |  |  | 1378 | 0.110 | 0.1588 | No |
| 105 | PRIM1 |  |  | 1411 | 0.108 | 0.1564 | No |
| 106 | TSPAN6 |  |  | 1430 | 0.107 | 0.1556 | No |
| 107 | CDCA7 |  |  | 1440 | 0.106 | 0.1559 | No |
| 108 | LIPG |  |  | 1468 | 0.105 | 0.1540 | No |
| 109 | PDPN |  |  | 1483 | 0.104 | 0.1537 | No |
| 110 | ANP32E |  |  | 1548 | 0.101 | 0.1473 | No |
| 111 | GAB1 |  |  | 1555 | 0.100 | 0.1479 | No |
| 112 | SSB |  |  | 1559 | 0.100 | 0.1489 | No |
| 113 | BAZ1A |  |  | 1603 | 0.097 | 0.1450 | No |
| 114 | HAT1 |  |  | 1609 | 0.097 | 0.1456 | No |
| 115 | ZBTB20 |  |  | 1632 | 0.095 | 0.1442 | No |
| 116 | MSI2 |  |  | 1640 | 0.095 | 0.1446 | No |
| 117 | PPIL4 |  |  | 1641 | 0.095 | 0.1459 | No |
| 118 | WRN |  |  | 1650 | 0.094 | 0.1461 | No |
| 119 | SOAT1 |  |  | 1674 | 0.093 | 0.1446 | No |
| 120 | FUT8 |  |  | 1685 | 0.093 | 0.1446 | No |
| 121 | BMS1 |  |  | 1698 | 0.092 | 0.1443 | No |
| 122 | ZKSCAN1 |  |  | 1711 | 0.091 | 0.1441 | No |
| 123 | OLIG2 |  |  | 1742 | 0.089 | 0.1416 | No |
| 124 | MCM2 |  |  | 1762 | 0.088 | 0.1405 | No |
| 125 | RIC3 |  |  | 1804 | 0.087 | 0.1367 | No |
| 126 | SCAF11 |  |  | 1822 | 0.085 | 0.1358 | No |
| 127 | MCM3 |  |  | 1918 | 0.080 | 0.1254 | No |
| 128 | PAICS |  |  | 1931 | 0.079 | 0.1250 | No |
| 129 | EXOSC8 |  |  | 1955 | 0.078 | 0.1232 | No |
| 130 | FAM161A |  |  | 1992 | 0.076 | 0.1199 | No |
| 131 | CBX5 |  |  | 2001 | 0.076 | 0.1199 | No |
| 132 | APBB2 |  |  | 2011 | 0.076 | 0.1198 | No |
| 133 | AXL |  |  | 2020 | 0.075 | 0.1199 | No |
| 134 | SRSF6 |  |  | 2027 | 0.075 | 0.1201 | No |
| 135 | BRIP1 |  |  | 2104 | 0.072 | 0.1119 | No |
| 136 | UHRF1 |  |  | 2136 | 0.071 | 0.1091 | No |
| 137 | CEP152 |  |  | 2138 | 0.071 | 0.1099 | No |
| 138 | TMEM18 |  |  | 2146 | 0.070 | 0.1100 | No |
| 139 | SRRM1 |  |  | 2157 | 0.070 | 0.1097 | No |
| 140 | HAUS6 |  |  | 2182 | 0.069 | 0.1077 | No |
| 141 | POLD3 |  |  | 2204 | 0.068 | 0.1061 | No |
| 142 | TRIM9 |  |  | 2215 | 0.068 | 0.1058 | No |
| 143 | NEDD1 |  |  | 2221 | 0.067 | 0.1060 | No |
| 144 | ZNF121 |  |  | 2230 | 0.067 | 0.1060 | No |
| 145 | MCM6 |  |  | 2237 | 0.067 | 0.1061 | No |
| 146 | ITGB3BP |  |  | 2263 | 0.066 | 0.1040 | No |
| 147 | NUP153 |  |  | 2274 | 0.065 | 0.1036 | No |
| 148 | POLA1 |  |  | 2330 | 0.063 | 0.0978 | No |
| 149 | MCM5 |  |  | 2344 | 0.063 | 0.0971 | No |
| 150 | RFTN2 |  |  | 2346 | 0.063 | 0.0978 | No |
| 151 | RFC1 |  |  | 2360 | 0.062 | 0.0970 | No |
| 152 | NOTCH3 |  |  | 2367 | 0.062 | 0.0971 | No |
| 153 | PARP1 |  |  | 2393 | 0.061 | 0.0949 | No |
| 154 | EGFR |  |  | 2416 | 0.060 | 0.0930 | No |
| 155 | SEPT10 |  |  | 2425 | 0.060 | 0.0929 | No |
| 156 | SRSF1 |  |  | 2450 | 0.059 | 0.0907 | No |
| 157 | CHEK1 |  |  | 2475 | 0.058 | 0.0886 | No |
| 158 | EIF3E |  |  | 2496 | 0.057 | 0.0869 | No |
| 159 | BARD1 |  |  | 2514 | 0.056 | 0.0856 | No |
| 160 | NPM1 |  |  | 2533 | 0.056 | 0.0842 | No |
| 161 | KIF22 |  |  | 2545 | 0.055 | 0.0836 | No |
| 162 | WDR76 |  |  | 2554 | 0.055 | 0.0834 | No |
| 163 | E2F7 |  |  | 2576 | 0.054 | 0.0815 | No |
| 164 | EXOSC9 |  |  | 2584 | 0.054 | 0.0814 | No |
| 165 | WEE1 |  |  | 2687 | 0.050 | 0.0698 | No |
| 166 | HSDL2 |  |  | 2688 | 0.050 | 0.0705 | No |
| 167 | CCNE2 |  |  | 2692 | 0.050 | 0.0708 | No |
| 168 | TRIM16 |  |  | 2693 | 0.050 | 0.0714 | No |
| 169 | DTL |  |  | 2697 | 0.050 | 0.0717 | No |
| 170 | HSPD1 |  |  | 2700 | 0.050 | 0.0721 | No |
| 171 | HAUS5 |  |  | 2707 | 0.050 | 0.0721 | No |
| 172 | RFC3 |  |  | 2708 | 0.050 | 0.0727 | No |
| 173 | ZEB1 |  |  | 2764 | 0.048 | 0.0667 | No |
| 174 | CTPS1 |  |  | 2789 | 0.047 | 0.0645 | No |
| 175 | WDHD1 |  |  | 2790 | 0.047 | 0.0651 | No |
| 176 | GPATCH4 |  |  | 2829 | 0.046 | 0.0611 | No |
| 177 | VRK1 |  |  | 2899 | 0.044 | 0.0534 | No |
| 178 | LDHB |  |  | 3031 | 0.040 | 0.0382 | No |
| 179 | ABAT |  |  | 3059 | 0.040 | 0.0354 | No |
| 180 | EIF2S1 |  |  | 3099 | 0.038 | 0.0312 | No |
| 181 | PARD3 |  |  | 3177 | 0.036 | 0.0225 | No |
| 182 | DIS3L |  |  | 3204 | 0.036 | 0.0198 | No |
| 183 | SNX4 |  |  | 3250 | 0.035 | 0.0148 | No |
| 184 | SRSF3 |  |  | 3283 | 0.034 | 0.0114 | No |
| 185 | MTCL1 |  |  | 3322 | 0.033 | 0.0073 | No |
| 186 | TLK1 |  |  | 3326 | 0.032 | 0.0074 | No |
| 187 | CDC7 |  |  | 3339 | 0.032 | 0.0063 | No |
| 188 | NSRP1 |  |  | 3341 | 0.032 | 0.0066 | No |
| 189 | EIF3M |  |  | 3357 | 0.032 | 0.0052 | No |
| 190 | IGSF11 |  |  | 3362 | 0.031 | 0.0052 | No |
| 191 | NUP43 |  |  | 3389 | 0.031 | 0.0024 | No |
| 192 | FEN1 |  |  | 3406 | 0.030 | 0.0009 | No |
| 193 | DCAF16 |  |  | 3421 | 0.030 | -0.0004 | No |
| 194 | PDLIM3 |  |  | 3422 | 0.030 | 0.0000 | No |
| 195 | SF3A3 |  |  | 3425 | 0.030 | 0.0002 | No |
| 196 | FAM111B |  |  | 3448 | 0.029 | -0.0021 | No |
| 197 | TIMELESS |  |  | 3497 | 0.028 | -0.0075 | No |
| 198 | ING5 |  |  | 3517 | 0.028 | -0.0094 | No |
| 199 | HNRNPA3 |  |  | 3532 | 0.027 | -0.0108 | No |
| 200 | CDC37 |  |  | 3535 | 0.027 | -0.0106 | No |
| 201 | TFDP2 |  |  | 3647 | 0.024 | -0.0237 | No |
| 202 | MNS1 |  |  | 3668 | 0.024 | -0.0258 | No |
| 203 | PAM |  |  | 3683 | 0.023 | -0.0271 | No |
| 204 | CEBPZ |  |  | 3687 | 0.023 | -0.0272 | No |
| 205 | TLE3 |  |  | 3709 | 0.022 | -0.0294 | No |
| 206 | ARHGAP5 |  |  | 3722 | 0.022 | -0.0306 | No |
| 207 | SKP2 |  |  | 3725 | 0.022 | -0.0305 | No |
| 208 | PIKFYVE |  |  | 3734 | 0.022 | -0.0312 | No |
| 209 | SALL1 |  |  | 3800 | 0.020 | -0.0388 | No |
| 210 | G2E3 |  |  | 3840 | 0.019 | -0.0432 | No |
| 211 | PRC1 |  |  | 3878 | 0.018 | -0.0474 | No |
| 212 | FOS |  |  | 3899 | 0.018 | -0.0496 | No |
| 213 | CCDC138 |  |  | 3960 | 0.016 | -0.0566 | No |
| 214 | FRMD5 |  |  | 3976 | 0.016 | -0.0582 | No |
| 215 | TRIM26 |  |  | 3984 | 0.016 | -0.0588 | No |
| 216 | PCNA |  |  | 3986 | 0.016 | -0.0588 | No |
| 217 | GNL3 |  |  | 4103 | 0.012 | -0.0725 | No |
| 218 | CUTA |  |  | 4115 | 0.012 | -0.0737 | No |
| 219 | PRPS2 |  |  | 4120 | 0.012 | -0.0740 | No |
| 220 | EGR1 |  |  | 4134 | 0.012 | -0.0754 | No |
| 221 | HNRNPF |  |  | 4135 | 0.012 | -0.0753 | No |
| 222 | C1orf35 |  |  | 4153 | 0.011 | -0.0772 | No |
| 223 | MBIP |  |  | 4173 | 0.010 | -0.0793 | No |
| 224 | GLCCI1 |  |  | 4180 | 0.010 | -0.0799 | No |
| 225 | SMAD5 |  |  | 4201 | 0.010 | -0.0822 | No |
| 226 | CRKL |  |  | 4237 | 0.009 | -0.0863 | No |
| 227 | CEP83 |  |  | 4339 | 0.006 | -0.0984 | No |
| 228 | PA2G4 |  |  | 4363 | 0.005 | -0.1011 | No |
| 229 | PDLIM5 |  |  | 4368 | 0.005 | -0.1015 | No |
| 230 | CENPK |  |  | 4433 | 0.003 | -0.1091 | No |
| 231 | PHF10 |  |  | 4472 | 0.002 | -0.1137 | No |
| 232 | MIS12 |  |  | 4478 | 0.002 | -0.1143 | No |
| 233 | SOX9 |  |  | 4503 | 0.001 | -0.1171 | No |
| 234 | LRR1 |  |  | 4533 | 0.000 | -0.1206 | No |
| 235 | ANXA7 |  |  | 4553 | -0.000 | -0.1229 | No |
| 236 | APEX1 |  |  | 4599 | -0.001 | -0.1283 | No |
| 237 | PRMT5 |  |  | 4610 | -0.002 | -0.1295 | No |
| 238 | MTA3 |  |  | 4718 | -0.004 | -0.1423 | No |
| 239 | FOXN3 |  |  | 4762 | -0.005 | -0.1474 | No |
| 240 | G3BP1 |  |  | 4777 | -0.005 | -0.1490 | No |
| 241 | POLI |  |  | 4858 | -0.007 | -0.1585 | No |
| 242 | DBF4 |  |  | 4926 | -0.009 | -0.1665 | No |
| 243 | UNG |  |  | 4979 | -0.010 | -0.1726 | No |
| 244 | C19orf48 |  |  | 4994 | -0.011 | -0.1741 | No |
| 245 | YAP1 |  |  | 5041 | -0.012 | -0.1795 | No |
| 246 | RPA2 |  |  | 5078 | -0.013 | -0.1836 | No |
| 247 | EPHB2 |  |  | 5132 | -0.014 | -0.1898 | No |
| 248 | PSD3 |  |  | 5168 | -0.015 | -0.1938 | No |
| 249 | LARP1B |  |  | 5252 | -0.017 | -0.2036 | No |
| 250 | LIN54 |  |  | 5277 | -0.018 | -0.2063 | No |
| 251 | TCF7L1 |  |  | 5378 | -0.020 | -0.2180 | No |
| 252 | BAG4 |  |  | 5413 | -0.021 | -0.2218 | No |
| 253 | HMGB2 |  |  | 5479 | -0.023 | -0.2293 | No |
| 254 | LYRM2 |  |  | 5614 | -0.028 | -0.2451 | No |
| 255 | PBX3 |  |  | 5653 | -0.029 | -0.2493 | No |
| 256 | FBL |  |  | 5681 | -0.029 | -0.2521 | No |
| 257 | TP53 |  |  | 5700 | -0.030 | -0.2539 | No |
| 258 | HDAC9 |  |  | 5716 | -0.030 | -0.2553 | No |
| 259 | NUP62 |  |  | 5739 | -0.031 | -0.2576 | No |
| 260 | NR1D2 |  |  | 5753 | -0.031 | -0.2587 | No |
| 261 | HES6 |  |  | 5805 | -0.033 | -0.2644 | No |
| 262 | TPM2 |  |  | 5823 | -0.034 | -0.2660 | No |
| 263 | CDK2 |  |  | 5871 | -0.035 | -0.2712 | No |
| 264 | C12orf29 |  |  | 5886 | -0.035 | -0.2724 | No |
| 265 | FAM114A1 |  |  | 5903 | -0.036 | -0.2739 | No |
| 266 | USP1 |  |  | 5963 | -0.038 | -0.2805 | No |
| 267 | DDAH1 |  |  | 6076 | -0.042 | -0.2934 | No |
| 268 | ALDH6A1 |  |  | 6109 | -0.043 | -0.2967 | No |
| 269 | TRMT10B |  |  | 6172 | -0.045 | -0.3036 | No |
| 270 | RFC5 |  |  | 6216 | -0.047 | -0.3081 | No |
| 271 | MDK |  |  | 6242 | -0.048 | -0.3105 | No |
| 272 | CDO1 |  |  | 6251 | -0.048 | -0.3108 | No |
| 273 | STK39 |  |  | 6252 | -0.048 | -0.3102 | No |
| 274 | ZC3HAV1 |  |  | 6254 | -0.049 | -0.3097 | No |
| 275 | C5orf30 |  |  | 6285 | -0.049 | -0.3126 | No |
| 276 | DKC1 |  |  | 6315 | -0.050 | -0.3155 | No |
| 277 | CORO1C |  |  | 6385 | -0.052 | -0.3231 | No |
| 278 | GMNN |  |  | 6392 | -0.052 | -0.3231 | No |
| 279 | RSL1D1 |  |  | 6425 | -0.054 | -0.3263 | No |
| 280 | PTPRN2 |  |  | 6469 | -0.055 | -0.3307 | No |
| 281 | QKI |  |  | 6517 | -0.057 | -0.3356 | No |
| 282 | ANXA5 |  |  | 6522 | -0.057 | -0.3354 | No |
| 283 | CKS2 |  |  | 6569 | -0.059 | -0.3401 | No |
| 284 | NUDT5 |  |  | 6594 | -0.060 | -0.3422 | No |
| 285 | DERA |  |  | 6623 | -0.062 | -0.3448 | No |
| 286 | HNRNPC |  |  | 6643 | -0.063 | -0.3462 | No |
| 287 | HNRNPD |  |  | 6663 | -0.064 | -0.3477 | No |
| 288 | TMSB15A |  |  | 6711 | -0.067 | -0.3525 | No |
| 289 | BBX |  |  | 6717 | -0.067 | -0.3522 | No |
| 290 | PRTFDC1 |  |  | 6760 | -0.069 | -0.3563 | No |
| 291 | ZFP36L2 |  |  | 6774 | -0.070 | -0.3570 | No |
| 292 | NAA50 |  |  | 6834 | -0.072 | -0.3631 | No |
| 293 | INPP5F |  |  | 6837 | -0.072 | -0.3624 | No |
| 294 | ZFP36L1 |  |  | 6893 | -0.076 | -0.3681 | No |
| 295 | HAUS1 |  |  | 6911 | -0.076 | -0.3691 | No |
| 296 | GLUL |  |  | 6961 | -0.079 | -0.3740 | No |
| 297 | CHIC2 |  |  | 6976 | -0.079 | -0.3746 | No |
| 298 | CLIC1 |  |  | 7041 | -0.084 | -0.3812 | No |
| 299 | DAXX |  |  | 7117 | -0.088 | -0.3891 | No |
| 300 | RBBP8 |  |  | 7128 | -0.089 | -0.3891 | No |
| 301 | MRPL24 |  |  | 7166 | -0.092 | -0.3924 | No |
| 302 | WWTR1 |  |  | 7187 | -0.093 | -0.3936 | No |
| 303 | CCDC59 |  |  | 7197 | -0.093 | -0.3934 | No |
| 304 | HMGB1 |  |  | 7231 | -0.096 | -0.3962 | No |
| 305 | TMA16 |  |  | 7244 | -0.096 | -0.3964 | No |
| 306 | ENO1 |  |  | 7257 | -0.097 | -0.3965 | No |
| 307 | C9orf3 |  |  | 7300 | -0.101 | -0.4003 | No |
| 308 | NUCKS1 |  |  | 7348 | -0.104 | -0.4046 | No |
| 309 | TIMM10 |  |  | 7349 | -0.104 | -0.4032 | No |
| 310 | MRPL45 |  |  | 7375 | -0.106 | -0.4048 | No |
| 311 | SNRPC |  |  | 7387 | -0.107 | -0.4047 | No |
| 312 | SNRPD1 |  |  | 7463 | -0.113 | -0.4123 | No |
| 313 | NAP1L1 |  |  | 7538 | -0.121 | -0.4196 | No |
| 314 | GINS2 |  |  | 7545 | -0.122 | -0.4187 | No |
| 315 | MMAB |  |  | 7557 | -0.123 | -0.4184 | No |
| 316 | CKS1B |  |  | 7569 | -0.124 | -0.4182 | No |
| 317 | SLC25A5 |  |  | 7582 | -0.126 | -0.4180 | No |
| 318 | DHFR |  |  | 7593 | -0.127 | -0.4175 | No |
| 319 | CDK6 |  |  | 7605 | -0.128 | -0.4172 | No |
| 320 | DUT |  |  | 7608 | -0.128 | -0.4157 | No |
| 321 | PRDX3 |  |  | 7649 | -0.132 | -0.4188 | No |
| 322 | CKB |  |  | 7659 | -0.134 | -0.4181 | No |
| 323 | SNRPB |  |  | 7671 | -0.136 | -0.4177 | No |
| 324 | TMEM106C |  |  | 7726 | -0.142 | -0.4223 | Yes |
| 325 | BIRC5 |  |  | 7735 | -0.144 | -0.4214 | Yes |
| 326 | RFC2 |  |  | 7739 | -0.144 | -0.4199 | Yes |
| 327 | AIF1L |  |  | 7768 | -0.148 | -0.4213 | Yes |
| 328 | CENPU |  |  | 7781 | -0.149 | -0.4208 | Yes |
| 329 | TYMS |  |  | 7792 | -0.150 | -0.4201 | Yes |
| 330 | NME1 |  |  | 7811 | -0.154 | -0.4202 | Yes |
| 331 | EEF1D |  |  | 7817 | -0.154 | -0.4188 | Yes |
| 332 | DPH3 |  |  | 7825 | -0.155 | -0.4176 | Yes |
| 333 | ARL4A |  |  | 7829 | -0.155 | -0.4160 | Yes |
| 334 | ALYREF |  |  | 7830 | -0.156 | -0.4139 | Yes |
| 335 | GLO1 |  |  | 7844 | -0.157 | -0.4134 | Yes |
| 336 | PRDX2 |  |  | 7855 | -0.159 | -0.4126 | Yes |
| 337 | HNRNPUL1 |  |  | 7889 | -0.164 | -0.4144 | Yes |
| 338 | HIST1H1C |  |  | 7917 | -0.170 | -0.4154 | Yes |
| 339 | H2AFZ |  |  | 7924 | -0.171 | -0.4139 | Yes |
| 340 | TOX3 |  |  | 7951 | -0.177 | -0.4147 | Yes |
| 341 | LSM6 |  |  | 7956 | -0.178 | -0.4129 | Yes |
| 342 | PABPC1 |  |  | 7960 | -0.179 | -0.4109 | Yes |
| 343 | RAN |  |  | 7979 | -0.181 | -0.4107 | Yes |
| 344 | TMEM263 |  |  | 7985 | -0.183 | -0.4089 | Yes |
| 345 | CHCHD3 |  |  | 7999 | -0.185 | -0.4081 | Yes |
| 346 | LITAF |  |  | 8015 | -0.188 | -0.4074 | Yes |
| 347 | H2AFY |  |  | 8098 | -0.203 | -0.4146 | Yes |
| 348 | ZFAND6 |  |  | 8115 | -0.207 | -0.4138 | Yes |
| 349 | RANBP1 |  |  | 8132 | -0.212 | -0.4130 | Yes |
| 350 | JUND |  |  | 8150 | -0.214 | -0.4122 | Yes |
| 351 | CDK4 |  |  | 8168 | -0.220 | -0.4114 | Yes |
| 352 | SET |  |  | 8175 | -0.221 | -0.4093 | Yes |
| 353 | RPL3 |  |  | 8247 | -0.238 | -0.4147 | Yes |
| 354 | GLTP |  |  | 8285 | -0.254 | -0.4158 | Yes |
| 355 | GSTP1 |  |  | 8296 | -0.257 | -0.4137 | Yes |
| 356 | RHOA |  |  | 8302 | -0.259 | -0.4109 | Yes |
| 357 | RPL15 |  |  | 8308 | -0.260 | -0.4081 | Yes |
| 358 | PEA15 |  |  | 8347 | -0.272 | -0.4091 | Yes |
| 359 | RPL5 |  |  | 8352 | -0.275 | -0.4060 | Yes |
| 360 | DEK |  |  | 8376 | -0.284 | -0.4051 | Yes |
| 361 | CNBP |  |  | 8379 | -0.286 | -0.4016 | Yes |
| 362 | SRI |  |  | 8395 | -0.292 | -0.3996 | Yes |
| 363 | RPS10 |  |  | 8404 | -0.297 | -0.3966 | Yes |
| 364 | PKM |  |  | 8420 | -0.301 | -0.3945 | Yes |
| 365 | RPL18 |  |  | 8424 | -0.304 | -0.3909 | Yes |
| 366 | RPL10 |  |  | 8426 | -0.304 | -0.3871 | Yes |
| 367 | COA4 |  |  | 8434 | -0.308 | -0.3839 | Yes |
| 368 | RPL13A |  |  | 8472 | -0.325 | -0.3841 | Yes |
| 369 | RPL7 |  |  | 8505 | -0.342 | -0.3835 | Yes |
| 370 | RPL10A |  |  | 8509 | -0.344 | -0.3793 | Yes |
| 371 | PTMA |  |  | 8523 | -0.348 | -0.3764 | Yes |
| 372 | CHCHD2 |  |  | 8525 | -0.348 | -0.3719 | Yes |
| 373 | NACA |  |  | 8534 | -0.354 | -0.3683 | Yes |
| 374 | RPL23 |  |  | 8539 | -0.357 | -0.3641 | Yes |
| 375 | RPL27A |  |  | 8541 | -0.358 | -0.3596 | Yes |
| 376 | RPS4X |  |  | 8542 | -0.359 | -0.3549 | Yes |
| 377 | YBX3 |  |  | 8545 | -0.362 | -0.3504 | Yes |
| 378 | LSM2 |  |  | 8558 | -0.371 | -0.3470 | Yes |
| 379 | HSPB1 |  |  | 8572 | -0.383 | -0.3436 | Yes |
| 380 | GNG5 |  |  | 8575 | -0.384 | -0.3388 | Yes |
| 381 | SNRPD2 |  |  | 8579 | -0.386 | -0.3341 | Yes |
| 382 | RPL21 |  |  | 8588 | -0.393 | -0.3299 | Yes |
| 383 | RPL7A |  |  | 8592 | -0.395 | -0.3251 | Yes |
| 384 | RPL9 |  |  | 8595 | -0.398 | -0.3202 | Yes |
| 385 | RPS3 |  |  | 8598 | -0.400 | -0.3152 | Yes |
| 386 | RPL8 |  |  | 8602 | -0.405 | -0.3103 | Yes |
| 387 | NUTF2 |  |  | 8604 | -0.405 | -0.3051 | Yes |
| 388 | RPL31 |  |  | 8606 | -0.406 | -0.2999 | Yes |
| 389 | GAPDH |  |  | 8615 | -0.418 | -0.2955 | Yes |
| 390 | RPS27A |  |  | 8620 | -0.422 | -0.2904 | Yes |
| 391 | PPIA |  |  | 8626 | -0.432 | -0.2854 | Yes |
| 392 | BTF3 |  |  | 8627 | -0.432 | -0.2797 | Yes |
| 393 | RAD23A |  |  | 8628 | -0.433 | -0.2741 | Yes |
| 394 | NME4 |  |  | 8630 | -0.434 | -0.2686 | Yes |
| 395 | ANAPC11 |  |  | 8633 | -0.439 | -0.2631 | Yes |
| 396 | RPL37A |  |  | 8636 | -0.440 | -0.2576 | Yes |
| 397 | RPS27 |  |  | 8638 | -0.441 | -0.2519 | Yes |
| 398 | RPL27 |  |  | 8639 | -0.441 | -0.2462 | Yes |
| 399 | RPL39 |  |  | 8640 | -0.443 | -0.2404 | Yes |
| 400 | HMGN2 |  |  | 8644 | -0.449 | -0.2349 | Yes |
| 401 | RPS16 |  |  | 8645 | -0.450 | -0.2290 | Yes |
| 402 | EEF1A1 |  |  | 8646 | -0.450 | -0.2232 | Yes |
| 403 | RPS19 |  |  | 8648 | -0.453 | -0.2174 | Yes |
| 404 | RPL23A |  |  | 8649 | -0.454 | -0.2114 | Yes |
| 405 | RPL4 |  |  | 8653 | -0.458 | -0.2058 | Yes |
| 406 | RPS18 |  |  | 8658 | -0.465 | -0.2002 | Yes |
| 407 | YBX1 |  |  | 8659 | -0.467 | -0.1941 | Yes |
| 408 | RPS25 |  |  | 8661 | -0.467 | -0.1882 | Yes |
| 409 | RPS28 |  |  | 8676 | -0.491 | -0.1835 | Yes |
| 410 | MZT2B |  |  | 8681 | -0.495 | -0.1775 | Yes |
| 411 | RPS3A |  |  | 8682 | -0.495 | -0.1710 | Yes |
| 412 | RPL13 |  |  | 8685 | -0.499 | -0.1648 | Yes |
| 413 | RPL6 |  |  | 8688 | -0.506 | -0.1584 | Yes |
| 414 | RPL36 |  |  | 8694 | -0.512 | -0.1523 | Yes |
| 415 | RPLP2 |  |  | 8697 | -0.515 | -0.1458 | Yes |
| 416 | RPS14 |  |  | 8700 | -0.518 | -0.1393 | Yes |
| 417 | RPLP0 |  |  | 8703 | -0.524 | -0.1327 | Yes |
| 418 | UQCRH |  |  | 8704 | -0.524 | -0.1259 | Yes |
| 419 | RPL18A |  |  | 8707 | -0.532 | -0.1192 | Yes |
| 420 | RPL29 |  |  | 8708 | -0.534 | -0.1122 | Yes |
| 421 | TPI1 |  |  | 8709 | -0.538 | -0.1052 | Yes |
| 422 | RPL35 |  |  | 8710 | -0.540 | -0.0981 | Yes |
| 423 | RPL32 |  |  | 8715 | -0.556 | -0.0914 | Yes |
| 424 | RPS6 |  |  | 8718 | -0.559 | -0.0843 | Yes |
| 425 | RPS13 |  |  | 8721 | -0.573 | -0.0771 | Yes |
| 426 | RPS15 |  |  | 8723 | -0.582 | -0.0696 | Yes |
| 427 | SERF2 |  |  | 8724 | -0.594 | -0.0618 | Yes |
| 428 | RPLP1 |  |  | 8728 | -0.606 | -0.0543 | Yes |
| 429 | RPS8 |  |  | 8730 | -0.621 | -0.0463 | Yes |
| 430 | RPSA |  |  | 8736 | -0.657 | -0.0383 | Yes |
| 431 | RPL12 |  |  | 8740 | -0.668 | -0.0300 | Yes |
| 432 | RPL41 |  |  | 8743 | -0.718 | -0.0208 | Yes |
| 433 | TUBA1B |  |  | 8745 | -0.733 | -0.0114 | Yes |
| 434 | TMSB4X |  |  | 8748 | -0.920 | 0.0004 | Yes |
Table: GSEA details [plain text format]

  

Fig 2: RICHARDS\_NOWAKOWSKI\_SCIENCE\_2017\_MGE\_RG2\_UPREG: Random ES distribution      
 Gene set null distribution of ES for **RICHARDS\_NOWAKOWSKI\_SCIENCE\_2017\_MGE\_RG2\_UPREG**
